# A Binational Overview of Reproductive Health Outcomes Among US Hispanic and Mexican Women in the Border Region

**DOI:** 10.5888/pcd10.130019

**Published:** 2013-08-15

**Authors:** Jill A. McDonald, Octavio Mojarro, Paul D. Sutton, Stephanie J. Ventura

**Affiliations:** Author Affiliations: Octavio Mojarro, CECOFIN SC, Mexico City, Mexico; Paul D. Sutton, Stephanie J. Ventura, Division of Vital Statistics, National Center for Health Statistics, Rockville, Maryland.

## Abstract

**Introduction:**

The US–Mexico border region has 15 million residents and 300,000 births annually. Reproductive health concerns have been identified on both sides of the border, but comparable information about reproductive health is not available. The objective of this study was to compare reproductive health indicators among populations in this region.

**Methods:**

We used 2009 US Hispanic and Mexican birth certificate data to compare births inside the border region, elsewhere within the border states, and in the United States and Mexico overall. We examined trends in total fertility and birth rates using birth data from 2000 through 2009 and intercensal population estimates.

**Results:**

Among women in the border region, US women had more lifetime births than Mexican women in 2009 (2.69 births vs 2.15 births) and throughout the decade. Birth rates in the group aged 15 to 19 years were high in both the US (73.8/1,000) and Mexican (86.7/1,000) border regions. Late or no prenatal care was nearly twice as prevalent in the border regions as in the nonborder regions of border states. Low birth weight and preterm and early-term birth were more prevalent in the US border than in the Mexican border region; US border rates were higher and Mexican rates were lower than their corresponding nonborder and national rates. We found some variations within border states.

**Conclusion:**

These findings constitute the first population-based information on the reproductive health of the entire Hispanic US–Mexico border population. Evidence of disparities warrants exploration at state and local levels. Teen pregnancy and inadequate prenatal care are shared problems in US–Mexico border communities and suggest an area for binational cooperation.

## Introduction

The United States–Mexico border stretches 2,000 miles from the Gulf of Mexico to the Pacific Ocean. The border region includes the area 100 km north (44 US counties) and 100 km south (80 Mexican *municipios*) of the border (1) (www.borderhealth.org/files/res_1741.pdf). The border population is approximately 15 million and is split almost equally between the United States and Mexico; in the United States, half of border residents are Hispanic (2). Nearly 300,000 births occur in the region annually (3,4). Whereas the border population is heterogeneous at the county/*municipio* level, it is medically underserved and has higher rates of poverty than the border state populations in the United States or Mexico (1,5). Perhaps in part because of these similarities, US and Mexican border communities are closely tied culturally, socially, and economically (6). High rates of fertility and immigration on both sides of the border have led to population projections of 23 million by the year 2030 (7).

Epidemiologic data for the border region are limited but suggest various reproductive health problems (8–11). Tabulated data from the US border region for 2008 have been published (10), but rates of reproductive health outcomes for the Mexican border region are not available. Lack of these data has been a barrier to the creation of binational health objectives (eg, Healthy Border 2020) (12). However, birth certificate records for Mexico have become available (4).

The Mexican birth certificate (13) does not include as much information as the US certificate (14), but the two share many demographic and outcome variables. Until recently, only birth estimates were available for Mexico (15). Our study objective was to provide an overview of reproductive health patterns in the US and Mexican border areas using birth certificate data. A secondary objective was to lay methodologic groundwork for future comparisons of the border region with border states and national rates using birth certificate data.

## Methods

This study describes fertility trends from 2000 through 2009 and then concentrates on registered births to residents of the United States and Mexico in 2009. US data were derived from special use files from the National Center for Health Statistics’ National Vital Statistics System (3). Three (California, New Mexico, and Texas) of the 4 border states had implemented the 2003 revision of the US Standard Certificate of Live Birth by 2009. Data for Arizona are based on the 1989 revision (16). Two data items were not available in a comparable format for Arizona: timing of prenatal care and educational attainment of the mother. Birth data for Mexico were derived from the birth certificates of registered births in 2009 (4).

Births were classified by county/*municipio* of maternal residence. Women reporting residence at the time of birth in the 44 border counties (US) and 80 *municipios* (Mexico) were defined as border residents. For the US analysis, we selected only births where the mother reported Hispanic ethnicity. We studied 3 US Hispanic and 3 Mexican birth populations: births in the border region (referred to simply as US border and Mexican border), births in the nonborder portions of border states (US nonborder and Mexican nonborder), and all births (US and Mexican).

The variables studied were maternal and infant indicators common to certificates in both countries (ie, demographic factors and birth outcomes). Determination of gestational age in the United States and Mexico was based on the interval between the first day of the mother’s last menstrual period and the date of birth, and on clinical assessment when last menstrual period was not available (13,14). For some variables (eg, maternal education) we collapsed response categories to create comparable measures.

We made side-by-side comparisons of the distributions of the indicators in the 6 study populations. Within the border region, we made some state-specific comparisons. Using birth data from 2000 through 2009 (3,15) and intercensal population estimates based on the 2000 and 2010 censuses in both countries (17,18), we calculated trends in total fertility rates. Birth rates were calculated for each 5-year age group between 10 and 49 years. We calculated general fertility rates for women aged 15 to 44 and 15 to 49.

## Results

### Fertility

**Figure 1:**
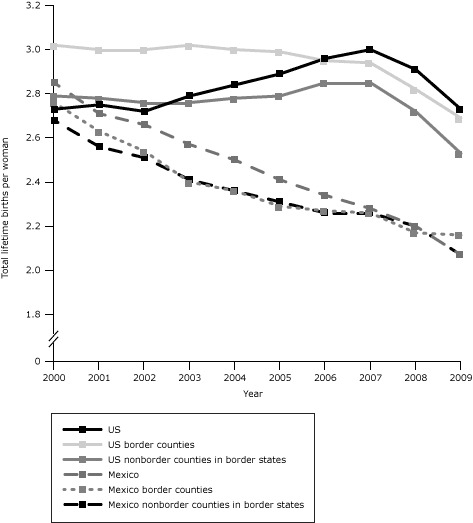
Trends in total fertility rates in the US Hispanic and Mexican populations overall, in the US and Mexican border regions, and the US and Mexican nonborder regions of border states, 2000–2009. Data sources for US and Mexican births, 2000–2009: National Center for Health Statistics (3), Sistema Nacional de Información en Salud (4), and Instituto Nacional de Estadística y Geografía (15). Data sources for population estimates: National Center for Health Statistics (17) and Instituto Nacional de Estadística y Geografía (18). **Table:** 

Mexican fertility rates on the border and elsewhere were similar to US Hispanic rates in 2000, but declined steadily thereafter (Figure 1). US rates were stable from 2000 through 2006 but declined steeply from 2006 through 2009. Despite the decline after 2006, by 2009 Hispanic women in the US border region had a total fertility rate 25% higher than women in the Mexican border region (Table 1).

Year	Total Lifetime Births Per Woman					
	US, %	US Border Counties, %	US Nonborder Counties in Border States, %	Mexico, %	Mexican Border Counties, %	Mexico Nonborder Counties in Border States, %
2000	2.73	3.02	2.79	2.85	2.76	2.68
2001	2.75	3.00	2.78	2.71	2.63	2.56
2002	2.72	3.00	2.76	2.66	2.54	2.51
2003	2.79	3.02	2.76	2.57	2.40	2.41
2004	2.84	3.00	2.78	2.50	2.36	2.36
2005	2.89	2.99	2.79	2.41	2.29	2.31
2006	2.96	2.95	2.85	2.34	2.27	2.26
2007	3.00	2.94	2.85	2.28	2.26	2.26
2008	2.91	2.82	2.72	2.20	2.17	2.20
2009	2.73	2.69	2.53	2.07	2.16	2.07

**Table 1:** Number of Births, Age-Specific Birth Rates,^a^ Total Fertility Rates,^b^ and General Fertility Rates^c^ in the US Hispanic and Mexican Populations in the United States and Mexico Overall, in the US and Mexican US–Mexico Border Regions, and in the US and Mexican Nonborder Regions of Border States, 2009^d^

Reproductive Outcome/Age	US Hispanic				Mexico			
	Border States			All States	Border States			All States
	Total	Border Region	Nonborder Region		Total	Border Region	Nonborder Region	
**No. of births, by maternal age group, y**								
All ages	526,789	84,474	442,315	999,548	357,809	148,820	208,989	2,046,209
10–14	1,156	211	945	2,073	2,903	1,269	1,634	16,704
15–19	77,712	13,962	63,750	136,263	74,096	32,768	41,328	406,377
20–24	146,130	24,054	122,076	274,726	103,143	45,603	57,540	603,316
25–29	140,178	22,057	118,121	270,641	87,885	34,967	52,918	502,232
30–34	99,697	15,216	84,481	195,729	58,619	22,179	36,440	327,910
35–39	50,269	7,310	42,959	97,261	26,173	10,007	16,166	154,606
40–44	11,041	1,586	9,455	21,638	4,538	1,856	2,682	31,424
45–49	578	72	506	1,168	338	133	205	2,773
**Age-specific birth rate, by maternal age group, y**								
10–14	1.0	1.1	0.9	1.0	3.1	3.1	3.0	3.0
15–19	66.1	73.8	64.6	63.6	81.0	86.7	77.0	72.5
20–24	141.7	151.1	140.0	140.1	120.2	133.7	111.3	116.6
25–29	135.6	145.4	134.0	134.3	106.0	106.1	105.9	105.8
30–34	101.1	105.2	100.4	100.8	70.5	65.2	74.1	71.3
35–39	52.1	49.1	52.6	52.5	32.6	30.1	34.4	35.8
40–44	12.9	11.6	13.1	13.2	6.7	6.6	6.7	8.4
45–49	0.8	0.6	0.8	0.8	0.6	0.6	0.6	0.9
**Total fertility rate**	2.56	2.69	2.53	2.53	2.09	2.15	2.05	2.06
**General fertility rate, by maternal age group, y**								
15–44	87.1	90.8	86.4	86.5	72.8	74.4	71.7	72.6
15–49	77.3	79.8	76.9	76.8	65.2	66.8	64.2	65.3

a Number of births per 1,000 women per age group.

b Calculated by using 5-year age groups from ages 10–14 through ages 45–49.

c Determined by using total number of births (regardless of maternal age) in numerator for both calculations.

d US data sources: US Census (17), National Vital Statistics System (3). Mexican data sources: Sistema Nacional de Información en Salud (4), Instituto Nacional de Estadística y Geografía (18).

In 2009, US border region births accounted for nearly 1 in 6 (16%) of all border state births, and border state births accounted for more than half of all US births. Births to Mexican border women accounted for 42% of births in the Mexican border states and 7% of births in Mexico (Table 1). The general and total fertility rates for US border women were about 5% higher than those for nonborder and US women. The general fertility rate for the US border region was 90.8 births per 1,000 women aged 15 to 44 in 2009; the nonborder rate was 86.4, and the US rate was 86.5. The total fertility rates were 2.69 births per lifetime for border women and 2.53 for nonborder and US women overall. US border birth rates among adolescents aged 15 to 19 were about 16% higher than those for US adolescents.

**Figure 2:**
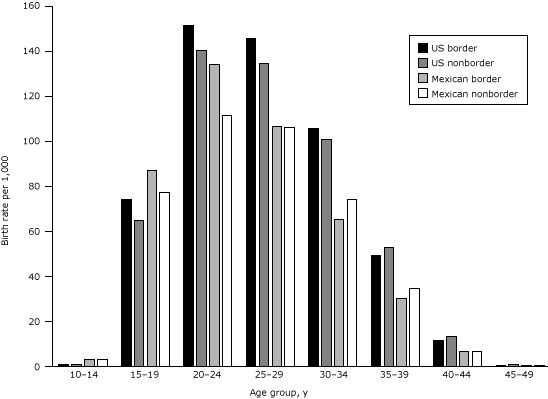
Age-specific birth rates per 1,000 women in the US Hispanic and Mexican populations in the border and nonborder regions of border states, 2009. Data sources for US and Mexican births: National Center for Health Statistics (3) and Sistema Nacional de Información en Salud (4). Data sources for population estimates: National Center for Health Statistics (17) and Instituto Nacional de Estadística y Geografía (18). **Table:** 

Among Mexican women in 2009, fertility rates were lower than the corresponding US rates. Mexican women in the border region had slightly higher general fertility (66.8/1,000 women aged 15–49) than nonborder women (64.2) or Mexican women (65.3). Especially high rates among border adolescents (86.7) and women aged 20 to 24 (133.7), which are 13% and 20% greater, respectively, than nonborder rates in these age groups, account for the border excess, similar to the pattern in the United States (Figure 2).

Age group, y	US Border States		Mexican Border States	
	US Border	US Nonborder	Mexican Border	Mexican Nonborder
10–14	1.1	0.9	3.1	3.0
15–19	73.8	64.6	86.7	77.0
20–24	151.1	140.0	133.7	111.3
25–29	145.4	134.0	106.1	105.9
30–34	105.2	100.4	65.2	74.1
35–39	49.1	52.6	30.1	34.4
40–44	11.6	13.1	6.6	6.7
45–49	0.6	0.8	0.6	0.6

### Birth outcomes and prenatal care

The proportions of preterm (<37 weeks gestation) and early-term (37–38 weeks) births were higher for US border than nonborder women (Table 2). By contrast, in Mexico, preterm and early-term births were less common among border than nonborder women. Preterm birth was more than twice as common among US border women (12.8%) as among Mexican border women (6.2%). Post-term births (≥42 weeks) were 3 times as prevalent among US border women (5.1%) as they were among Mexican border women (1.6%). Birth weight distributions were consistent with results for gestational age in the United States and Mexico. In the United States, 7.4% of border births were low birth weight (<2,500 g) compared with 6.7% of nonborder births, and 5.8% were high birth weight (≥4,000 g) compared with 7.3% of nonborder births. On the other hand, low birth weight was less common among Mexican border births (6.3%) than among nonborder (7.0%) or Mexican births (8.5%) and high birth weight was more common (7.3%, border region; 6.2%, nonborder region; 4.3%, Mexico). The rates of multiple births (twins, triplets, and higher-order multiples) were similar for Hispanic women in the 3 geographic areas of the United States, and occurrence was somewhat more common than in Mexico.

**Table 2:** Proportions^a^ of Births According to Birth Outcomes and Prenatal Care Indicators in the US Hispanic and Mexican Populations in the United States and Mexico Overall, in the US and Mexican US–Mexico Border Regions, and in the US and Mexican Nonborder Regions of the Border States, 2009^b^

Outcome	US Hispanic			Mexico		
	Border States		All States	Border States		All States
	Border Region	Nonborder Region		Border Region	Nonborder Region	
**Gestational age, wk**						
<37	12.8	11.4	12.0	6.2	7.0	6.6
37–41	82.1	83.3	82.2	92.2	91.8	92.0
37–38	33.4	28.9	28.3	24.2	30.0	26.9
≥42	5.1	5.3	5.8	1.6	1.2	1.4
Total	100.0	100.0	100.0	100.0	100.0	100.0
**Birthweight, g**						
<2500	7.4	6.7	6.9	6.3	7.0	8.5
≥4000	5.8	7.3	7.1	7.3	6.2	4.3
**Multiple birth**	2.4	2.2	2.3	1.5	1.8	1.5
**Late or no prenatal care, by maternal age group, y^c^**						
<20	17.6	11.8	12.3	14.3	8.8	10.0
20–34	13.5	7.2	8.4	8.9	5.0	6.7
≥35	12.0	5.8	6.7	8.0	3.6	6.2
All ages	14.0	7.7	8.4	10.1	5.7	7.4
**Mean no. of prenatal care visits by maternal age group, y**						
<20	10.0	10.5	10.3	6.7	6.8	6.7
20–34	10.6	11.2	10.9	7.6	7.9	7.4
≥35	10.9	11.7	11.4	8.0	8.4	7.8
All ages	10.5	11.2	10.9	7.4	7.7	7.3

a All values are percentages unless otherwise indicated.

b US data sources: National Vital Statistics System (3). Mexican data sources: Sistema Nacional de Información en Salud (4).

c Data from the following states were not included because of incomparability between the 1989 and 2003 revisions of US Standard Certificate of Live Birth: Alabama, Alaska, Arizona, Arkansas, Connecticut, Hawaii, Illinois, Louisiana, Maine, Maryland, Massachusetts, Minnesota, Mississippi, Missouri, Nevada, New Jersey, North Carolina, Oklahoma, Rhode Island, Virginia, West Virginia, Wisconsin, and Washington, DC.

Among Hispanic women giving birth in US border states, women in the border region (14.0%) were almost twice as likely as women in the nonborder region (7.7%) to begin prenatal care in the third trimester or have no care at all. These rates were higher than the corresponding rates for Mexican women; but, like US border women, their Mexican counterparts more frequently received late or no prenatal care than Mexican nonborder region women (10.1% vs 5.7%). For all US and Mexican subgroups, the average number of prenatal care visits by age was inversely related to the prevalence of late or no prenatal care. Among women who received care, US women had a greater mean number of visits, regardless of subgroup.

### Other maternal and delivery characteristics

Among US Hispanic births in the border region, nonborder region, and all states, fewer than half occurred among women born in Mexico (Table 3). Comparing the 3 US geographic subgroups, border region women most frequently had at least a high school education. Among Mexican women, educational attainment in the border region was similar to that in Mexico overall; about one-third in each group had an 8th grade education or less, and one-quarter in each group had completed high school. Mexican nonborder women were better educated than other Mexican women. US border women were more frequently married than their nonborder counterparts and US women. Mexican border women, on the other hand, were less frequently married than nonborder or Mexican women.

**Table 3:** Proportions of Births, by Selected Maternal and Delivery Characteristics in the US Hispanic and Mexican Populations Overall, in the US and Mexican Border Regions, and in the US and Mexican Nonborder Regions of Border States, 2009^a^

Characteristic	US Hispanic			Mexico		
	Border States		All States	Border States		All States
	Border Region	Nonborder Region		Border Region	Nonborder Region	
**Maternal Characteristics**						
**Born in Mexico**	42.8	43.2	39.2	99.6	99.8	99.7
**Education^b^**						
≤8th grade/*secundaria incompleta*	10.4	14.4	15.4	31.8	22.7	33.8
>8th grade/*secundaria completa*	89.6	85.6	84.6	68.2	77.3	66.2
High school incomplete/ preparatoria incompleta	27.4	27.6	26.6	42.3	41.3	37.6
≥High school/≥preparatoria completa	62.3	57.9	58.0	25.8	36.0	28.6
**Marital status**						
Married	51.4	47.3	46.8	41.1	57.2	49.3
Unmarried	48.6	52.7	53.2	58.9	42.8	50.7
**Parity**						
1	36.1	34.7	35.3	36.9	39.3	39.6
≥4	15.0	16.1	15.0	12.4	9.8	12.2
**Delivery Characteristics**						
**Cesarean delivery**	37.9	30.9	31.6	43.1	46.3	44.5
**Hospital or clinic birth**	99.8	99.8	99.7	99.8	99.8	97.8
**Birth attendant**						
Physician	91.3	93.5	91.1	99.6	97.7	96.9
All other, including nurse and midwife	8.7	6.5	8.9	0.4	2.3	3.1

Abbreviation: CI, confidence interval.

a US data sources: National Vital Statistics System (3). Mexican data sources: Sistema Nacional de Información en Salud (4).

b Data from the following states were not included because of incomparability between the 1989 and 2003 revisions of the US Standard Certificate of Live Birth: Alabama, Alaska, Arizona, Arkansas, Connecticut, District of Columbia, Hawaii, Illinois, Louisiana, Maine, Maryland, Massachusetts, Minnesota, Mississippi, Missouri, Nevada, New Jersey, North Carolina, Oklahoma, Rhode Island, Virginia, West Virginia, and Wisconsin. In addition, data for smoking during pregnancy were not available for Florida, Georgia, or Michigan.

Cesarean delivery was more prevalent among US border births than among US nonborder or US births. The percentage of births by cesarean was generally higher in Mexican than in US subgroups but slightly lower among Mexican border than Mexican nonborder or Mexican women. Virtually all US and Mexican women delivered their babies in a hospital or clinic. In Mexico, physician-attended birth was the norm whereas in the US almost 1 in 11 Hispanic births was not attended by a physician.

### US and Mexican border states

In the United States, higher proportions of preterm and early-term births in the border counties compared with the nonborder counties were limited to Texas and New Mexico; the patterns were reversed in Arizona and California (Table 4). The prevalence of high birth weight was greatest in California. In California and New Mexico, border counties surpassed nonborder counties in the rate of high birth weight. The disparity in cesarean birth rates between border and nonborder counties was seen in all border states, but it was especially notable in Texas: 44% of border county births were cesarean deliveries compared with 30% of nonborder county births. Similarly, the high prevalence of late or no prenatal care in border compared with nonborder counties was apparent in all 3 US states for which data were available. Omitting San Diego County in California, which had better outcomes on almost all measures, increased the level of disparity between US border and nonborder births for most outcomes.

**Table 4:** Proportions of US Hispanic and Mexican Births With Selected Outcomes in Border and Nonborder Regions, by State, 2009^a^

Outcome	Total Births, n	Preterm (<37 wk), %	Early Term (37–38 wk), %	Birth Weight ≥4000 g, %	Cesarean Birth,%	Late or No Prenatal Care, %
**US Border States**						
All US border states	526,789	11.6	29.6	7.0	32.1	8.7
All border counties	84,474	12.8	33.4	5.8	37.9	14.0
All border counties except San Diego County, California	64,745	13.9	35.9	4.8	39.8	17.2
All nonborder counties	442,315	11.4	28.9	7.3	30.9	7.7
**Texas**	201,227	13.1	32.2	5.9	33.5	14.7
Border counties	48,098	14.7	38.9	4.2	43.5	17.3
Nonborder counties	153,129	12.6	30.2	6.5	30.3	13.8
**New Mexico**	16,158	12.6	26.2	4.8	22.8	9.6
Border counties	3,727	13.6	26.7	5.4	27.7	15.5
Nonborder counties	12,431	12.3	26.0	4.6	21.3	7.5
**Arizona**	39,168	12.8	30.2	6.8	25.5	NA
Border counties	10,073	11.2	27.5	6.7	26.0	NA
Nonborder counties	29,095	13.3	31.2	6.8	25.3	NA
**California**	270,236	10.3	27.7	8.0	32.5	4.1
Border counties	22,576	9.3	25.6	8.8	33.1	6.9
Nonborder counties	247,660	10.4	27.9	7.9	32.5	3.8
**Mexican Border States**						
All Mexico border states	357,809	6.7	27.6	6.7	45.0	7.5
All border *municipios*	148,820	6.2	24.2	7.3	43.1	10.1
All nonborder *municipios*	208,989	7.0	30.0	6.2	46.3	5.7
**Tamaulipas**	64,947	6.1	26.9	6.4	48.8	7.3
Border *municipios*	36,146	5.9	26.7	6.6	49.8	8.7
Nonborder *municipios*	28,801	6.4	27.2	6.1	47.6	5.4
**Nuevo Leon**	75,044	7.5	34.8	5.1	51.1	4.2
Border *municipios*	1,687	5.3	25.2	6.2	43.7	3.3
Nonborder *municipios*	73,357	7.6	35.0	5.1	51.3	4.3
**Coahuila**	54,336	6.0	27.8	5.9	43.7	9.5
Border *municipios*	13,524	6.5	23.3	6.9	43.5	10.9
Nonborder *municipios*	40,812	5.8	29.2	5.6	43.7	9.1
**Chihuahua**	59,214	6.7	23.4	6.1	36.7	7.8
Border *municipios*	26,492	5.7	20.6	6.8	34.3	10.2
Nonborder *municipios*	32,722	7.5	25.8	5.5	38.7	5.9
**Sonora**	46,894	7.2	24.8	10.1	43.7	5.8
Border *municipios*	13,597	6.6	21.1	9.5	40.9	8.9
Nonborder *municipios*	33,297	7.4	26.3	10.3	44.8	4.5
**Baja California^b^**	57,374	6.5	25.2	7.5	43.3	11.3

Abbreviation: NA, data not available.

a US data sources: National Vital Statistics System (3). Mexican data sources: Sistema Nacional de Información en Salud (4).

b Baja California *municipios* are all within the border region.

In Mexican border states, preterm birth occurred less frequently in border than nonborder *municipios* in each state except Coahuila. Consistent with this finding, high birth weight was more frequent among border than nonborder babies in all states but Sonora, where prevalence was especially high in both border and nonborder *municipios*. The higher prevalence of cesarean birth in the nonborder *municipios* was the reverse of the US pattern and was shared by all states except Tamaulipas and Coahuila. Late or no prenatal care was more prevalent in the border than the nonborder *municipios* of every state except Nuevo Leon.

## Discussion

This study’s findings constitute the first population-based information on the reproductive health of the entire Hispanic US–Mexico border population. It describes populations that live in close proximity and share a common genetic and cultural heritage but still differ in important ways from one another and from nonborder populations. The differences noted likely reflect various factors, including socioeconomic status, local and national policies, and health care systems.

Trends in total fertility rates on the US side of the border are consistent with economic improvement in the standard of living for US Hispanics during the 2000s and the sharp downturn in income with the recession of the US economy beginning in 2008 (19). In contrast, total fertility rates in Mexico, which exceeded those of US Hispanics in 2000, have declined rapidly in all populations, with the most likely explanation being the success of Mexican family planning programs (20).

General fertility rates among US and Mexican border women were only slightly higher than those of their US and Mexican nonborder counterparts. The largest differences across age groups between border and nonborder regions were among younger women: women younger than 30 in the United States and younger than 25 in Mexico. Birth certificate coverage may be more complete in Mexican border communities, which are more urban and provide greater access to birth facilities than more rural communities elsewhere in Mexico (5). If true, however, more complete coverage along the border would have increased rates for all age groups and not just those of the youngest women. Therefore, the higher fertility rates along the border are unlikely to be an artifact of better birth certificate coverage.

In the United States, characteristics of nonborder and US births overall were similar, but the prevalence of preterm and early-term births and low birth weight was higher among border births. The cesarean delivery rate was also highest in the US border region at 38%, well above recommendations of 5% to 15% (21) and well above the US Hispanic rate, which is similar to the rate for all US women (32.9%) (22). The cesarean rate in Texas border counties (43.5%) and rates in most Mexican areas of at least 40% are of particular note. The Mexican border had fewer preterm, early-term, and low-weight births and more high-weight births than elsewhere in Mexico; cesarean delivery rates in the Mexican border were slightly lower. Low and high birth weight are associated with the development of obesity and diabetes (23), which are especially prevalent in the border region (2,11,24). Higher rates of late or no prenatal care among US and Mexican border births than births in the interior of either country may reflect border women receiving care on both sides of the border (25) with resulting disruption in insurance coverage and incompleteness of prenatal records. Fewer prenatal visits among Mexican women may also be influenced by national norms in Mexico that recommend fewer visits for low-risk women (26) than do guidelines followed in the United States (27).

Mothers in US border counties had higher levels of education than other US Hispanic mothers, perhaps reflecting longer residence in the established Hispanic communities of the border region. Mothers in Mexican border *municipios*, in contrast, had education levels comparable to Mexico overall, perhaps reflecting migration to the border for jobs in the *maquila* industry there (28). Mothers in the nonborder regions of Mexican border states were better educated, consistent with the lower poverty rates found in nonborder *municipios* (5). The higher percentage of physician-attended deliveries in Mexico than in the United States might be in part an artifact of incomplete reporting of births out of hospital attended by nonphysicians.

State-specific analysis showed that the border populations are not homogeneous. In the United States, Texas border counties and Texas overall had the highest proportions of adverse outcomes. California tended to have better outcomes, but this reflects the affluence of San Diego, which is in the US border region, and contrasts strongly with the less affluent counties to the east. In Mexico, the state of Nuevo Leon had nearly complete receipt of prenatal care, whereas in the state of Baja California coverage was almost as poor as that in US border counties. These interstate differences might be due to differences in income levels, Medicaid coverage (in the United States), or other local health policies.

This is the first study to 1) make use of recently available Mexican birth certificate files, thereby allowing the first comparison of births throughout the border region of the United States and Mexico, 2) compare birth outcomes in individual states within and across the border, and 3) compare Hispanic births in the border and nonborder regions of the border states. Study results may help binational advisory groups and health authorities in the region to identify and implement the best reproductive health practices found on either side of the border.

This study had several limitations. The completeness of Mexican data and the accuracy of some of the birth certificate information are still being assessed. However, at the time of our analysis, we found 3.5% fewer 2009 births estimated to have occurred in Mexico’s border region *municipios* (15) than the 148,820 registered in the birth certificate file (4). Roughly 95% of births in Mexico now take place in hospitals and clinics (15); hospitals, clinics, and midwives are all required to submit birth certificates (13). Misclassification of country of residence in the census and on the birth certificate among undocumented women in the United States and Mexico might affect birth rates, but the direction of the bias is unknown. US states have slowly been implementing the 2003 revision of the birth certificate, and some US registrars or hospitals are still inexperienced with some new topics or revised items in this revision. The US Hispanic population is heterogeneous. Border Hispanics are almost exclusively Mexican, whereas only two-thirds of US Hispanics are of Mexican descent (22); this factor likely influenced some differences between border states and the United States overall.

In addition, numerous birth certificate variables are only collected in 1 of the 2 countries. For example, Mexico alone collects information on type of hospital and administration of bacillus Calmette-Guerin vaccine, whereas the US alone collects information on maternal morbidity and weight gain during pregnancy. One variable, payment source for delivery, collected in both countries, was not yet available from the US National Vital Statistics System and is not included in this report. Such “asymmetrical” information is not useful in a binational comparison of birth outcomes, but it will be useful in future explorations of reproductive health outcomes and in future attempts to link birth outcomes to adult diseases. For example, information on maternal smoking, prepregnancy body mass index, diabetes, and hypertension could all be exploited in both countries if collected in a standardized way on birth certificates.

This report suggests numerous action steps for public health in the region. Overall priorities on the US side of the border include reducing the numbers of teenage births, preterm and early-term births, low birth weights, cesarean deliveries, and women receiving late or no prenatal care. On the Mexico side, priorities include reducing the numbers of teenage births, cesarean deliveries (which are exceptionally high throughout Mexico), and women receiving late or no prenatal care. Given the heterogeneity of the border population, reproductive health outcomes need to be analyzed not just for the border region as a whole but for the border regions of each state. At least among Hispanics, the border regions are not like the border states or the national Hispanic population, and one state’s border region is not like another’s. Local health departments and community groups can use these data to help set local priorities. Differences in reproductive health outcomes for contiguous US and Mexican border communities might offer clues to factors driving poorer outcomes on one side of the border. Conversely, adjacent border communities with the same reproductive health priorities can collaborate on joint actions to address the needs of a binational population that spends time on both sides of the border (6).

Finally, the US–Mexico border regions share a host of other health problems among adults that are related to their shared genetic makeup and behavioral patterns. For example, US Hispanics along the border and their Mexican neighbors both have high rates of obesity, diabetes, and cervical cancer (29,30). Future analyses of comparable administrative data sets (eg, mortality data) might reveal the full extent and determinants of these chronic diseases and the factors that contribute to their excess. Sources of such comparable data may already be available, and parallel behavioral risk factor surveys might now be developed. Behavioral surveys currently exist on both sides of the border (30–32), but no attempt has been made to improve their comparability. Such standardization could enhance binational collaboration on various health problems across the lifespan.
